# The influence of microinjection parameters on cell survival and procedure efficiency

**DOI:** 10.1016/j.mex.2023.102107

**Published:** 2023-03-06

**Authors:** Joanna Hajduk, Konrad Szajna, Bartosz Lisowski, Zenon Rajfur

**Affiliations:** a Doctoral School of Exact and Natural Sciences, Jagiellonian University, Łojasiewicza 11, Krakow 30-348, Poland; b Faculty of Physics, Astronomy and Applied Computer Science, Jagiellonian University, Łojasiewicza 11, Krakow 30-348, Poland; c Faculty of Pharmacy, Jagiellonian University Medical College, Medyczna 9, Kraków 30-688, Poland; d Jagiellonian Center of Biomedical Imaging, Jagiellonian University, Kraków 30-348, Poland

**Keywords:** Microinjection, Manual, Semi-automatic, Cell culture, Fibroblasts, Adherent cells manual and semi-automatic microinjection

## Abstract

Microinjection is a method commonly used to deliver various substances into cells. The procedure is performed on a widefield microscope stage using fine glass needle to penetrate the cell membrane. Microinjection can be carried out using a manual or semi-automatic mode. For commercially available equipment currently reported microinjection success rate and cell viability are relatively low (around 50% for both indicators). Here, for the first time, we systematically show how the microinjection effectiveness and cell viability are influenced by needle diameter and chosen microinjection mode. We found that manual mode entailed a higher injection rate, reducing cell viability at the same time. The reduction in needle diameter caused a significant increase in cell survival rate (from 43 to 73% for manual mode and from 58% to 86% for semi-automatic mode) and did not affect significantly the success rate. Our findings will help optimize this method in the context of cell biology research.

• This study shows how to improve microinjection parameters, such as procedure efficiency and cell survival rate, for commercially available equipment.
• Manual mode, in comparison with semi-automatic mode, results in higher microinjection efficiency, but lower cell survival rate.
• The increase in micropipette diameter causes lower cell viability and a higher microinjection success rate.

## Graphical abstract

**Figure:**
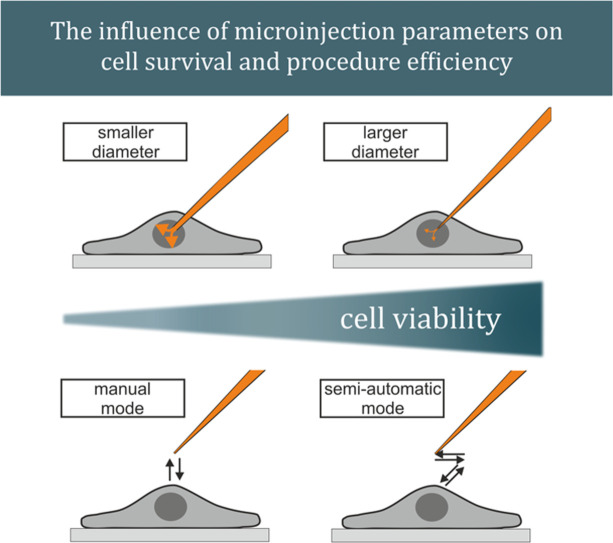

Specifications table

**Table:** 

Subject area:	Agricultural and Biological Sciences
More specific subject area:	Cell biology
Name of your method:	Adherent cells manual and semi-automatic microinjection
Name and reference of original method:	Not applicable
Resource availability:	Equipment used in this study is commercially available:
	• micromanipulator InjectMan® NI 2 (Eppendorf) • microinjector FemtoJet® (Eppendorf) • micropipette Puller P-97 (Sutter Instrument)

## Method details

### Background

Microinjection is a precise method of delivering dissolved or suspended substances, such as enzymes, ions, dyes, deoxyribonucleic acids, and proteins, into cell nucleus or cytoplasm. The injection procedure is usually performed on the microscope stage, using a fine glass needle (tip diameter usually less than 1 µm) to puncture cell membrane. Microinjection is frequently used in molecular biology, cytology, pharmacology and genetic engineering studies [1,2].

One of the advantages of microinjection is the possibility of studying single cell immediately after substance delivery. It can be used for a wide range of cell lines and primary cultures. It requires only small amounts of delivered substances, which is important when using expensive or difficult-to-prepare products. There are also some disadvantages to the microinjection technique. First, cells can be injected only one at a time, which means that during a single experiment only a limited number of cells can be studied. It also requires a certain amount of experimenter experience and specialized equipment [3]. Microinjection may also cause mechanical damage to cells [2].

**Fig. 1:**
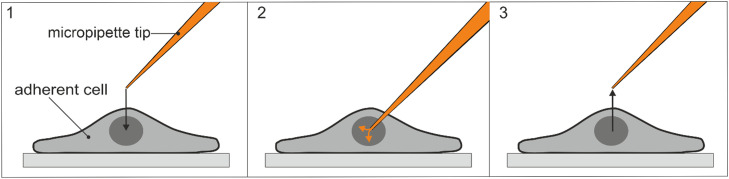
Manual microinjection into an adherent cell.

Microinjection can be performed using a manual or semi-automatic system. To perform manual microinjection, the tip of the micropipette is centered right above the cell compartment intended for injection. Then, the needle is manually lowered along the y-axis until it enters the cell (Fig. 1). The solution is injected into the cell with an application of previously chosen pressure. In this mode, the user cannot choose a precise injection time and has to manually control it, by lifting the needle from the cell at the right time. The manual system is usually considered slower than the semi-automatic one - it allows to inject about 100–200 cells in 30 min, compared to 200–300 cells injected in a typical semi-automatic system. However, if the user has adequate experience, manual microinjection can be as fast and reproducible as semi-automatic microinjection [3].

**Fig. 2:**
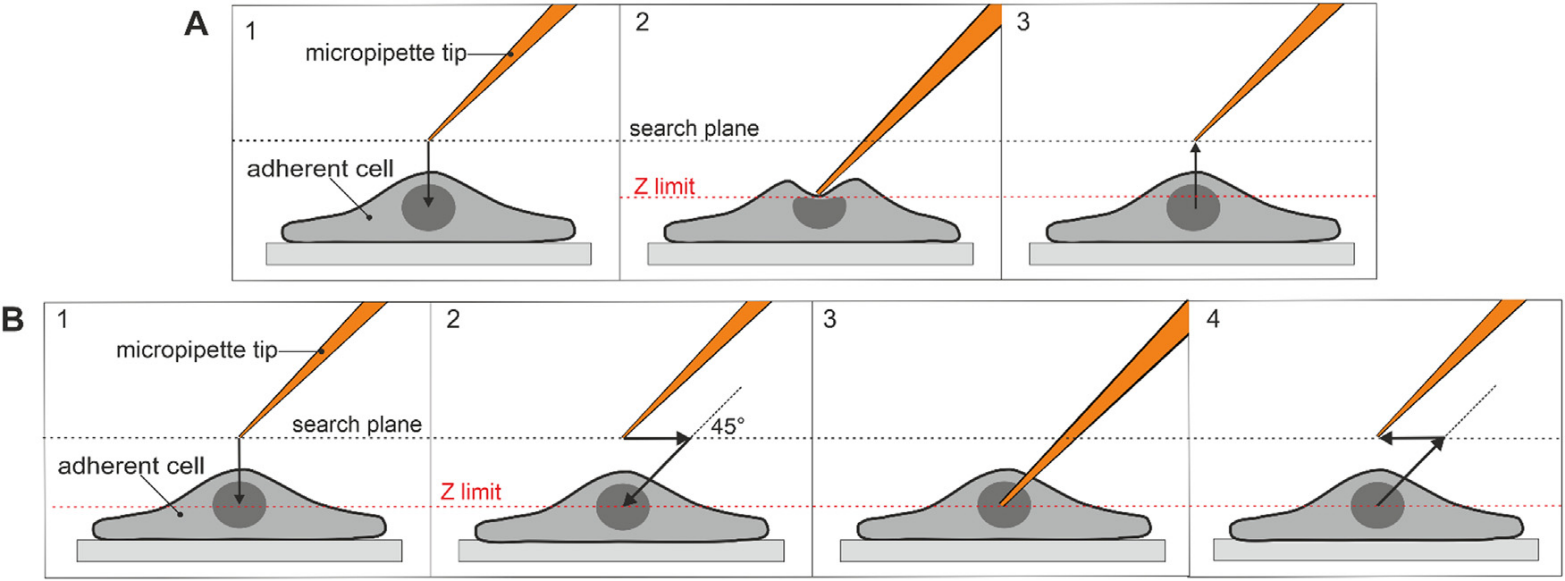
Semi-automatic microinjection. (A) Setting of the Z-axis limit for semi-automatic microinjection. (B) Semi-automatic microinjection into an adherent cell.

**Fig. 3:**
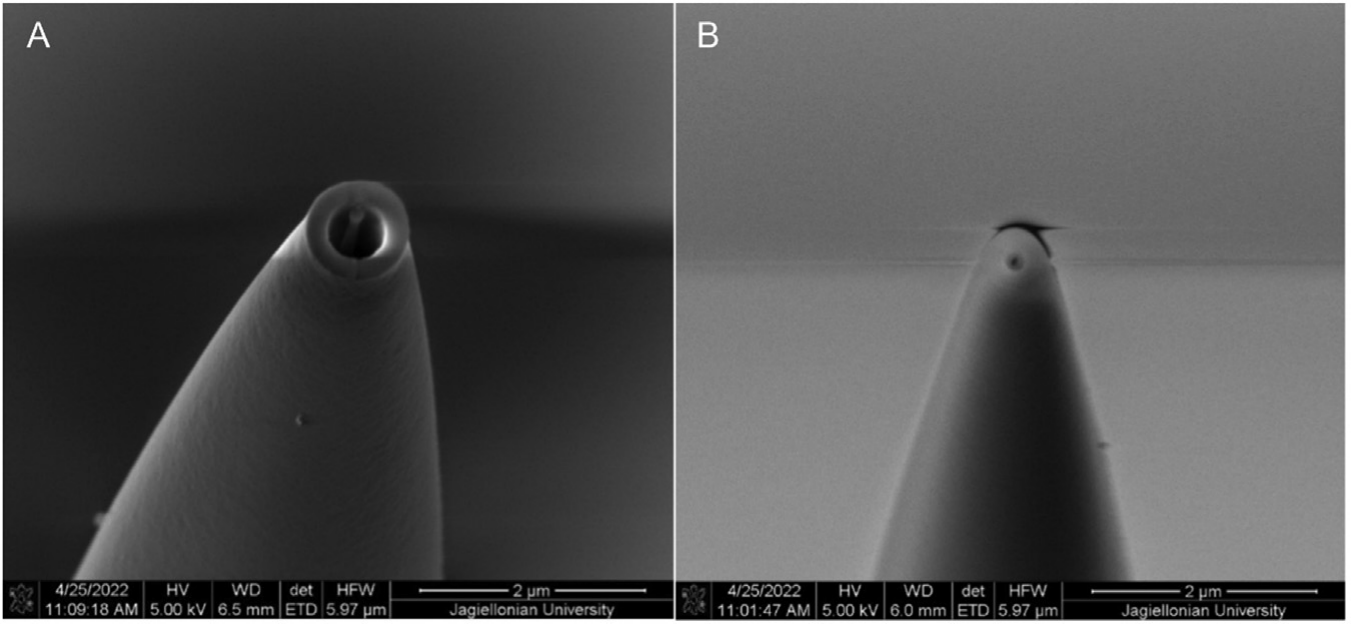
Representative SEM microscope images of micropipette tips. Micropipette type I tip (left) and micropipette type II tip (right).

**Fig. 4:**
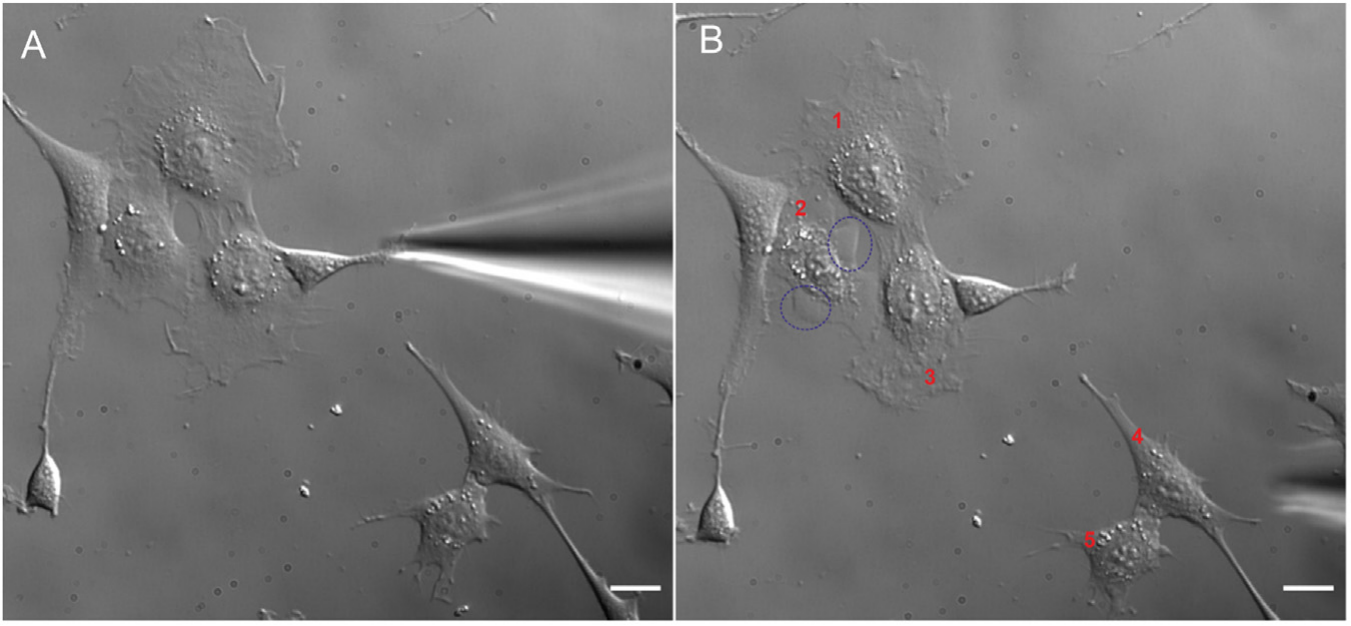
Cells before (left) and after (right) penetration test. Injected cells are marked with red numbers. Blebs produced by cells as a result of high pressure are surrounded by a blue dotted line. The scale bar represents 20 μm.

In semi-automatic microinjection, the user first chooses the injection height (Z-axis limit or Z-limit for brevity), which is set by slowly lowering the pipette until it touches the cell membrane (Fig. 2A-2). This causes a small deformation on the cell surface that becomes visible under the microscope. The user can always adjust the Z-limit later. After the Z-axis limit is set, the tip of the micropipette should be centered above the cell compartment that is to be injected (Fig. 2A-3). Then, the injection is performed automatically: the pipette moves first to the right and then axially down (usually at the 45° angle to the dish surface) until it reaches the Z-limit (Fig. 2B-2). Once there, the injection pressure that was previously set is applied for the chosen time (Fig. 2B-3). After the procedure, needle automatically returns to its previous location (Fig. 2B-4) [1,3].

The volume of injected solution depends on microinjection time, capillary tip opening diameter and flow of injected solution (which in turn depends on solution type and microinjection pressure) [3,4]. Chow et al. showed that injection volume increases linearly with increasing injection pressure. Similarly, increasing the injection time leads to a linear increase in volume [5].

Semi-automatic microinjection is believed to cause less damage to cells than manual injection because it minimizes the mechanical pressure applied to cells upon axial injection. In addition, there is a smaller probability that cellular components attach to the micropipette. Usually, the semi-automatic method is also described as faster than the manual method [1,3,6].

Little research has been conducted on micropipette fabrication and its effect on microinjection success rate and cell viability. There are some reports of increased viability as a result of the decrease in outer tip diameter, but to the best of our knowledge, the confirming results have not been published [7].

This paper compares and evaluates manual and semi-automatic microinjection techniques in order to increase microinjection success rate and cell survival rate. We found that semi-automatic microinjection is better in terms of cells viability, although it shows lower effectiveness. Using two types of micropipettes with significantly different diameters, we show that tip diameter strongly affects the viability of injected MEF 3T3 cells.

## Materials

### Cell culture

The MEF 3T3 cell line (Mouse Embryonic Fibroblasts, ATCC CRL-1658) was cultured in DMEM Low Glucose medium (Dulbecco's Modified Eagle Medium; Biowest, France) supplemented with 10% FBS (Fetal Bovine Serum; ThermoFisher Scientific, USA) and 1% PS (penicillin and streptomycin; Biowest, France) at 37 °C, 5% CO_2_. Cells used in these experiments had between third and thirteenth passage.

One day prior to the experiments, cells were seeded in 35 mm cell imaging glass bottom dishes (thickness #1.5) (Cellvis, USA). Cells that were placed overnight in the incubator for survival test, were seeded in a 35 mm gridded glass bottom dish (Cellvis, USA). Cells were imagined in FluoroBrite^TM^ DMEM medium (ThermoFisher Scientific, USA) supplemented with 2% FBS, 1% PS, 1% 200 mM L-glutamine (ThermoFisher Scientific, USA) and 1% sodium pyruvate 100 mM (EuroClone, Italy).

### Reagents

For microinjection, we used rhodamine B isothiocyanate dextran of molecular weight 70 kDa (Sigma-Aldrich, USA). Powdered dextran was dissolved in filtered sterile PBS (phosphate-buffered saline) at a concentration of 10 mg/ml. Dextran used for microinjection was diluted to a concentration of 2.5 mg/ml in PBS. Right before the experiment, dextran was centrifuged for 5 min at 201 RCF to remove any possible contaminations or dextran clusters.

## Fluorescence microscopy

Live cell imaging was performed using Zeiss Axio Observer Z1 inverted fluorescence microscope with a 40x, 0.55 NA objective and Hamamatsu ORCA-Flash 4.0 camera. Heating Insert P Lab-Tek™ S (Pecon, Germany) together with TempModule S (Pecon, Germany) and CO_2_ Module S (Pecon, Germany) provided the right conditions for live cell imaging: 37 °C and 5% CO_2_. Rhodamine-labeled dextran was observed using the standard Zeiss filter cube 43 HE, while fluorescein-labeled dextran was observed with the filter cube 38 HE.

## Micropipette fabrication

The micropipettes used for microinjection were prepared from glass capillaries using Micropipette Puller P-97 (Sutter Instrument, USA). Borosilicate glass capillaries (outside diameter: 1.0 mm, inside: 0.5 mm) were purchased from Sutter Instruments. The puller was equipped with a box-type heating filament.

Puller heats the glass capillary with a heating platinum filament. Then, the capillary is pulled with two handles to produce tapered glass needles^3^. It is possible to control the diameter and length of the pipette tip by adjusting several parameters: heat, pull, velocity, time/delay, and pressure. The heat value results from the amount of electrical current applied to the filament. It is determined by running a ramp test, which informs about the minimal heat needed to melt the glass. Usually, we use heat values from Ramp-10 to Ramp+15. The lower the heat, the shorter and wider the tips. The pull value refers to the strength with which both ends of the filament are pulled after the glass has softened - the higher the pull, the smaller the tip. The velocity value determines the separation rate of the puller bar - higher velocity produces a longer pipette taper. There is the possibility to choose between time mode and delay mode. The time parameter controls the duration of air cooling; it is typically set between 150 (75 ms) and 250 (125 ms), depending on the type of filament and capillaries. The delay mode is used for longer cooling (up to 300 ms). It is the delay time between the end of the glass capillary heating and the initiation of the pull process. The longer delay value, the shorter pipette is produced. The pressure parameter adjusts the air pressure that cools the capillary. A pressure of 500 is recommended for thick wall capillaries (like those used in the described experiment) [8,9]. In Table 1 two sets of parameters used for micropipettes fabrication (hence: type I with bigger tip and type II with smaller tip micropipettes) are listed, as well as minimal compensation pressure p_c_ for each of them.

**Table 1:** Parameter settings used for micropipette fabrication. The Ramp value is the result of the ramp test and informs about the minimum amount of heat required to melt the glass capillary. Ramp = 508. Pc is the minimal compensation pressure for 70 kDa dextran.

Parameter	Heat	Pull	Velocity	Delay	Pressure	P_c_ [hPa]
Micropipette type I	Ramp-10	30	30	250	500	≈ 10–15
Micropipette type II	Ramp	50	50	150	500	≈ 90–120

To precisely measure inner and outer diameters of micropipettes we employed the electron microscopy technique. They were imaged by scanning electron microscopy with a Quanta 3D FEG microscope (FEI, USA) (Fig. 3). The images were obtained based on a secondary electron signal, detected by the Everhart-Thornley detector, using an electron beam energy of 5 keV and a current of 53 pA. Measurement was carried out under high vacuum conditions of 6*10^−4^ Pa. Micropipettes were imaged at an angle of 74°.

Seven needles of each kind (pulled separately) were measured to ensure homogenity in needle preparation. The results of the measurements are summarized in Table 2. The finer needles are less homogeneous than the wider ones. For both micropipette types outer diameter is less uniform than inner diameter.

**Table 2:** Results of the micropipette tip diameter measurements. The results in this table are shown as mean ± standard error. Number of measured micropipettes *n*=7. One of the measured micropipettes type II was not taken into consideration due to irregular shape of the micropipette opening.

Micropipette type	OTD [nm]	ITD [nm]
Micropipette type I	920.8 ± 24.0	523.6 ± 10.8
Micropipette type II	508.5 ± 85.9	207.3 ± 35.4

## Microinjection system

Microinjection equipment was from Eppendorf (Germany) and included: micromanipulator InjectMan® NI 2, microinjector FemtoJet® and motor module placed on the microscope adapter. Injection was performed on a wide-field fluorescence microscope stage.

The microinjector controls the injection parameters such as: injection pressure, time, and compensation pressure. The injection pressure is the pressure applied to inject liquid from the microneedle into the cell. Injection time determines how long the liquid is injected. Compensation pressure is a pressure maintained in the capillary to prevent liquid flow from the dish due to capillary action. The correct value of the compensation pressure is the minimum pressure at which a constant, small outflow of dextran from the needle is observed.

The micromanipulator allows for precise capillary manipulation in all three dimensions with the joystick. It is used to set the limit on the z-axis, perform manual microinjection, and execute semi-automatic injection [10].

## Microinjection procedure

The micropipettes were loaded with 2 µl of dextran using Microloader™ tips (Eppendorf, Germany). A loaded micropipette was placed in the grip head and attached to the capillary holder and then placed in microscope light path and lowered with a micromanipulator until it was directly above cells (under 10x objective observation). Then the magnification was increased to 40x. A minimal p_c_ was set to ensure a small dextran leakage from the pipette. Cells were then injected using a manual or semi-automatic technique. In semi-automatic microinjection, the injection pressure p_i_ was usually twice that of the compensation pressure p_c_, while the angle of the needle was set at 45°. In manual mode, the injection pressure could usually be lower than in semi-automatic mode. The injection pressure was visually determined by microinjecting a few cells with different pressure. The goal was to find the minimum pressure at which the microinjection would be successful.

## Microinjection tests

Three tests were performed to evaluate microinjection: penetration, injection, and survival. For each test, at least three separate samples from different cell passages were used. The number of cells tested was summed up.

### Penetration test

Penetration test was performed to check whether the needle penetrates through the cell membrane. The penetration rate is defined as the ratio of cells with successfully penetrated membranes to the number of attempted injections. It is possible that needle deforms cell without puncturing the membrane and entering the cell which can be difficult to observe the difference under an optical microscope [2]. This test is important to determine whether the Z-limit has been set appropriately.

To verify the effectiveness of penetration, we injected cells at much higher pressure than needed. If the procedure resulted in a clear cell response (such as visible ‘penetration wave’ shown on Supplementary Video 1) or cell membrane eruption, we assumed that the membrane was penetrated successfully. Table 3 shows the penetration test settings for both types of micropipettes.

**Table 3:** Penetration, injection and survival test settings.

Test	Penetration test				Injection and survival test			
Micropipette	Micropipette type I		Micropipette type II		Micropipette type I		Micropipette type II	
Method	Manual	Semi-automatic	Manual	Semi-automatic	Manual	Semi-automatic	Manual	Semi-automatic
Compensation pressure p_c_ [hPa]	10–15	10–15	90–100	90–100	10–15	10–15	90–100	90–100
Injection pressure p_i_ [hPa]	120–150	150–180	350–400	450–500	10–30	20–50	100–120	150–180
Injection time t_i_ [s]	-	0.1	-	0.2	-	0.1	-	0.2

Fig. 4 shows cells before (left) and after (right) the penetration test (in manual mode). It can be seen that injection with such high pressure affected cells, causing their morphological changes. Cell number two, for instance, produced blebs (surrounded by a blue dotted line), while cell number three shrunk. Cell reaction to penetration test is clearly visible in Video 1 attached in the supplementary materials. There, we can clearly see the ‘propagation wave’ of injected material through the cell to lamellipodia.

### Injection test

Another measured parameter was the injection success rate. It may be defined as the ratio of cells successfully injected to the number of injection attempts.

**Fig. 5:**
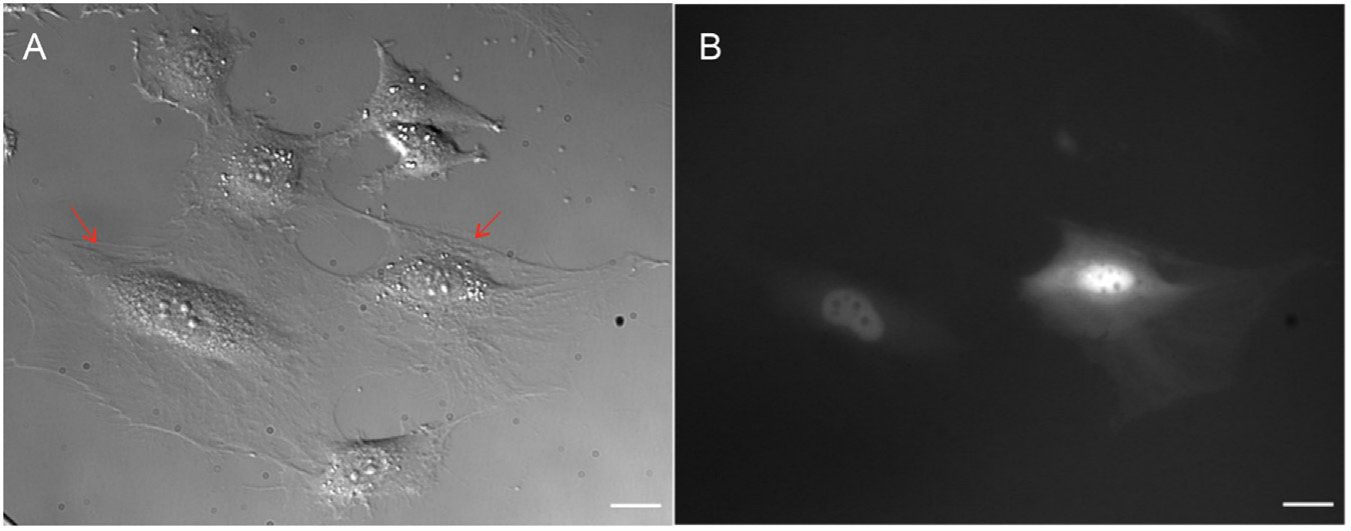
MEF 3T3 cells immediately after microinjection with rhodamine B labeled dextran 70 kDa. Image with DIC contrast (left) and fluorescent image (right). Red arrows indicate successfully injected cells. The scale bar represents 20 μm.

To perform an injection test, the appropriate pressure and Z-limit (if necessary) were established. The pressure had to be high enough for dextran to enter the cell but not too high to damage it. Both the Z-limit and the injection pressure had to be adjusted during the experiment. After the procedure, the injection success rate was assessed with a fluorescence microscope. The injection test settings are shown in Table 3. An example of cells subjected to the microinjection test is shown in Fig. 5.

### Survival test

Survival rate is defined as the ratio of injected cells that survived microinjection to the number of cells successfully injected. The survival test requires several hours of observation to determine the long-term cell viability. Immediately after microinjection, the shape and adhesion status of the injected cell can change in reaction to the mechanical pressure of the needle. Microinjection may cause some visible damage to the cell, but, after some time, cell sometimes returns to its previous shape. It may take few hours for the cell to regenerate, which explains the need for long observation [2]. Video 2 in the supplementary materials presents round and shrunken cell 15 min after microinjection. However, after one hour, the cell has spread and dextran is visible inside it.

After microinjection, all cells were observed for at least 4 h. Few randomly selected cells from a group injected semi-automatically with type I needle were also checked after 20 h to verify long-term viability. For those cells non-quantitative analysis was performed for illustrative purposes. Pressure settings and injection time were the same as in injection test (Table 3).

**Fig. 6:**
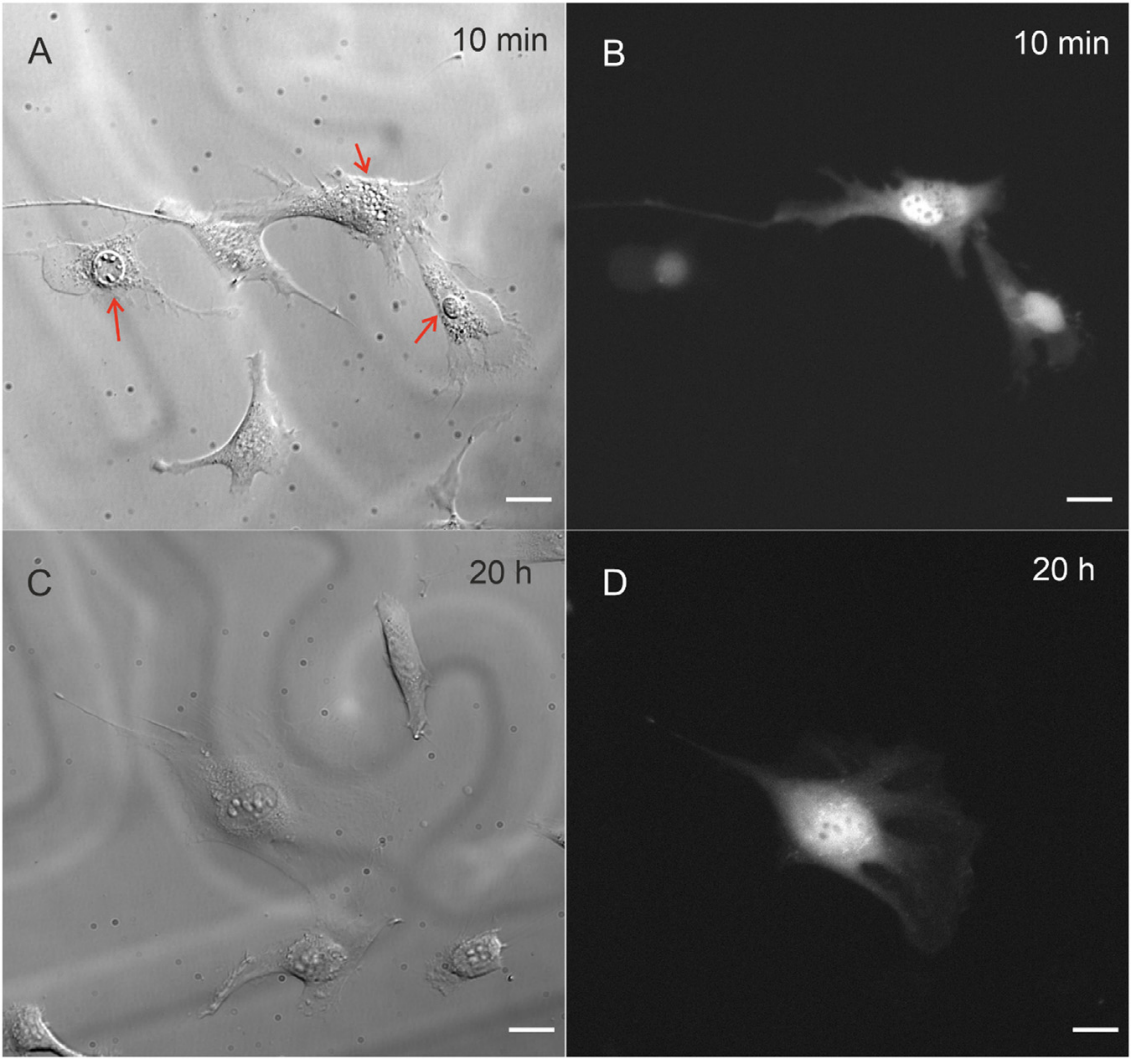
Representative image of survival test results. Cells 10 min after microinjection: DIC contrast (A) and fluorescence image (B). Red arrows mark injected cells. The same cells 20 h after microinjection: DIC contrast (C) (fluorescence picture (D). Shades in the background are engraved grids, used to identify cells positions at different times. The scale bar represents 20 μm.

An example of a survival test is shown in Fig. 6. Cells are shown 10 min and 20 h after microinjection. Three injected cells are visible in the field of view (Fig. 6A and B), of which two did not survive; they formed blebs a few minutes after injection. One of the injected cells survived 20 h (Fig. 6C and D).

### Statistical analysis

N-1 Chi-Square test was used to compare between groups. Differences were considered to be statistically significant when p-values were <0.05. Per each test 60–87 cells in total were examined. Data with no statistically significant differences between groups was indicated by” n.s.”.

## Results and conclusions

**Fig. 7:**
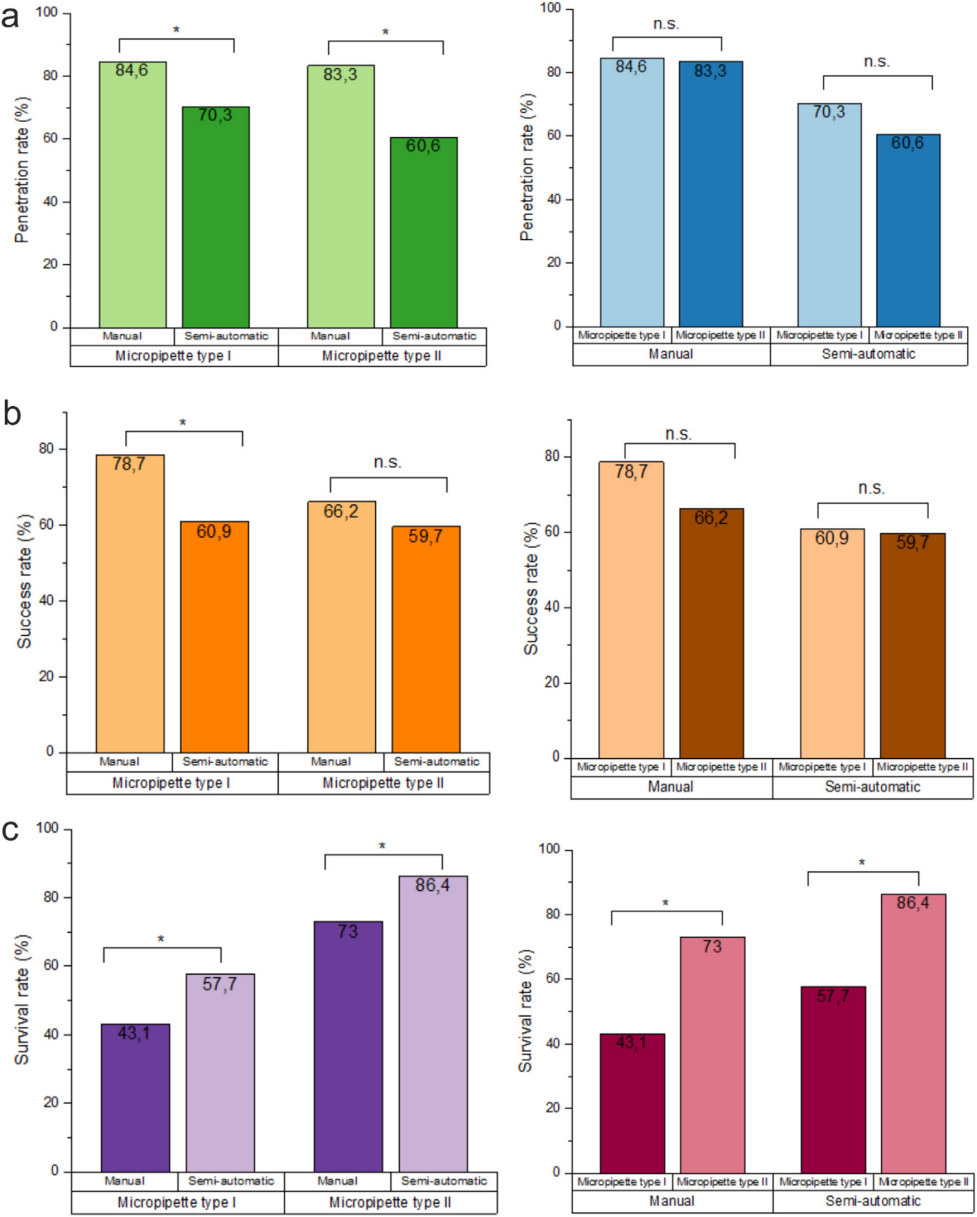
Microinjection penetration, injection and survival tests results. (a) Percentage of cells with successfully penetrated membranes. Sample sizes *n*=60–74 cells. Comparison of manual and semi-automatic mode penetration rate (left) and type I and II micropipettes penetration rate (right). (b) Percentage of successfully injected cells. Sample sizes *n*=61–87 cells. Comparison of manual and semi-automatic mode microinjection success rate (left) and type I and II micropipettes success rate (right). (b) Percentage of viable cells 4 h after microinjection. Sample sizes *n*=63–66 cells. Comparison of manual and semi-automatic mode cell survival rate (left) and type I and II micropipettes survival rate (right). “n.s.” indicates that difference between groups is not statistically significant, * statistically significant differences with a *p*-value <0.05 (N-1 Chi-Square test).

The manual injection penetration rate was significantly higher for both micropipette types (Fig. 7). The main reason is that in the manual mode user can control and adjust the depth of the microinjection during the process, while in the semi-automatic mode, the movement is performed automatically to the previously selected limit. It means that in the case of different cell sizes, once set, the Z-limit may not fit all of them. Penetration success rate depends also on the microinjection speed correctly set (in semi-automatic mode). Viigipuu et al. (2004) reported a semi-automatic microinjection penetration rate of 87% for MCF-7 and SH-SY5Y cells [2]. We reported lower penetration rate for semi-automatic mode: 70.3% and 60.6%, respectively for type I and type II micropipettes, which could be caused for example by insufficient needle speed settings. Penetration rate also can be affected by cell height heterogeneity or alignment of dish surface.

One of the main issues that affect the microinjection success rate is needle clogging. It may occur due to: (1) incorrect needle manipulation, such as dragging the needle over cells or an incorrect setting of injection depth, which may detach cells and attach them to the needle; (2) attaching parts of the cell membrane to the micropipette (most often due to insufficient injection pressure); (3) inadequate compensation pressure that aids the unwanted reverse flow of medium into the capillary; (4) the presence of contaminations in the sample; (5) an overly long time span between filling the capillary and inserting the needle into the medium [4]. Therefore, the success of the whole process depends on the correct setting of Z-limit (in semi-automatic mode) and microinjection pressure setting.

For type I micropipette, successful delivery of dextran was achieved in 78.7% for manual mode and in 60.9% for semi-automatic mode (statistically significant, *p* < 0.05) (Fig. 7). For type II micropipette, the test values obtained were 66.2% and 59.7% respectively (not-significant difference) (Fig. 7). The success rate was slightly lower for type II needle (not-significant differences), probably because dextran flow was reduced due to a smaller micropipette ITD. For both types of micropipettes, the manual mode allows for the possibility of achieving a higher success rate than it would be in the case of utilizing the semi-automatic mode. These differences are probably due to a higher penetration rate for semi-automatic microinjection.

The microinjection success rate we achieved is higher than the score reported by Lim et al. (2011) for the same Eppendorf equipment. They obtained a semi-automatic injection success rate of 49.2 ± 4.2% and 50.8 ± 4.6% (depending on pressure), using a pre-pulled capillary, also purchased from Eppendorf (outer tip diameter approximately 0.5 μm) [12]. Viigipuu and Kallio reported an injection success rate of 49% for semi-automatic injection of MCF-7 cells. They used a custom-made microinjection system [2]. Chow et al. also described the development of a new microinjection system, with an achieved success rate of 88.0 and 58.5% for HFF and hESC-VCM cells [6].

The microinjection survival rate was measured to determine the damage caused to cells by the procedure itself. We showed that the lower micropipette tip diameter is, the higher cell viability is. For wider tips (ITD of about 516 nm), we achieved a survival rate of 43.1% for manual mode and 57.7% for semi-automatic mode. For tips with decreased inner diameter (approximately 157 nm), the survival test results were significantly higher: 73.0% and 86.4%, respectively (Fig. 7). Our results for semi-automatic mode are better than those achieved by Lim et al. (2011) (50.98% ± 4.67% and 49.72 ± 5.48%), especially for the pipette with a smaller tip [11].The viability obtained by different groups ranged from 55 to 81% [6,7,12]. Davis et al. did not report a significant difference between the 24 h viability of CD34+ cells injected manually and semi-automatically with Oregon Green dextran (they were 55 ± 8.5% and 56 ± 5.2% respectively) [7].

Cells injected manually exhibited significantly lower viability, what indicates that moving needle at a 45° angle with higher speed requires lower mechanical pressure to puncture the cell membrane. That reduces cell damage. Increased cell survival rate using needles with smaller tips proves that proper injected sample volume and pressure are crucial for cell survival. Decreasing the ITD of the micropipette reduced the volume of dextran that flows into the cell during injection, lowering the possibility of cell membrane damage.

In conclusion, we show here that the diameter of the micropipette tip strongly affects cell viability. Cell survival rate is higher for a smaller tip diameter, what probably is caused by a lower flow of material and a decrease in damage to the cell membrane during injection. We present this effect for two significantly different types of needles. Effectiveness of microinjection and cells viability also depend on chosen injection mode. Manual mode results in higher efficiency, but at the same time, lower survival rate. It also requires more time to inject the same number of cells. On the other hand, semi-automatic microinjection is quicker but less effective. However, it results in higher viability than manual one.

These results can be helpful for researchers who use microinjection technique in their experimental work to obtain better results, repeatability, and higher cell viability. It can also be a basis for a broader analysis of the influence of needle shape and diameter on microinjection results, which may be beneficial while using different types of delivered material.

### Ethics statements

None.

## CRediT authorship contribution statement

**Joanna Hajduk:** Writing – original draft, Investigation, Visualization. **Konrad Szajna:** Investigation. **Bartosz Lisowski:** Conceptualization, Funding acquisition, Writing – review & editing. **Zenon Rajfur:** Supervision, Conceptualization, Writing – review & editing.

## Declaration of Competing Interest

The authors declare that they have no known competing financial interests or personal relationships that could have appeared to influence the work reported in this paper.

## Data Availability

Data will be made available on request.

## Appendix Supplementary materials

Video 1. The example of penetration test. Adherent cell was injected manually with high pressure. The penetration of presented cell was successful because ‘propagation wave’ of injected material is visible. Image, video 1

Video 2. Time-lapse recording of cell injected with fluorescent dextran. The video starts 15 min after microinjection. Cell was observed for one hour. Image, video 2
